# Mental Distress and Smoking in Relation to Cardiovascular Mortality in the United States Population

**DOI:** 10.1002/brb3.71590

**Published:** 2026-07-09

**Authors:** Shengpang Wang, Yu‐Jun Xiong, Xiang‐Da Meng, Tian Lv, Jingjing Lou

**Affiliations:** ^1^ Department of Psychiatry Shaoxing Seventh People's Hospital Shaoxing Zhejiang People's Republic of China; ^2^ Department of Gastroenterology, Beijing Hospital, National Center For Gerontology; National Clinical Research Center For Gerontology; The Key Laboratory of Geriatrics of NHC; Institute of Geriatric Medicine Chinese Academy of Medical Sciences Beijing People's Republic of China; ^3^ Department of Hernia and Abdominal Wall Surgery Peking University People's Hospital Beijing People's Republic of China; ^4^ Department of Neurology Zhuji Affiliated Hospital of Wenzhou Medical University Zhuji People's Republic of China

**Keywords:** county health, CVD mortality, mental distress, nationwide study, smoking

## Abstract

**Background:**

Frequent mental distress and adult smoking are prevalent population‐level risk indicators that may contribute to geographic and sociodemographic disparities in cardiovascular disease (CVD) mortality in the United States. Their county‐level associations with CVD mortality and major subtypes remain incompletely understood.

**Methods:**

This nationwide ecological study included data from 3069 US counties (2016–2020). Exposure data were sourced from the County Health Rankings & Roadmaps program; mortality data were obtained from the CDC WONDER. Age‐adjusted mortality rates (AAMRs) were calculated for overall CVD and key subtypes. Counties were stratified into quartiles for each exposure. Associations were assessed using generalized linear models with quasi‐Poisson distributions.

**Results:**

Counties with higher levels of frequent mental distress and adult smoking demonstrated progressively elevated CVD mortality. Total CVD AAMR increased across distress quartiles from 365.93 to 509.24 per 100,000, with the strongest associations for acute myocardial infarction (AMI) and ischemic heart disease (IHD). Adult smoking showed a similar gradient, with total CVD mortality rising from 376.45 to 532.50 per 100,000 and the steepest increase again observed for AMI. These patterns persisted after multivariable adjustment and were most pronounced among adults aged 45–64 years, with variation across racial and urbanization strata.

**Conclusions:**

Higher county‐level prevalence of frequent mental distress and adult smoking was associated with higher cardiovascular mortality after adjustment for measured county‐level covariates, particularly from AMI and IHD. The findings reveal substantial geographic and sociodemographic disparities, supporting integrated public health strategies that concurrently address mental health, tobacco control, and social determinants to mitigate population‐level cardiovascular risk.

## Graphical abstract

County‐level mental distress was associated with increased cardiovascular mortality in minimally adjusted models. After full adjustment, these associations were largely attenuated, with significant associations persisting only at lower exposure levels for selected ischemic cardiovascular outcomes.

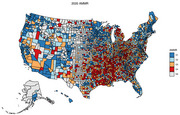

## 1 Introduction

Cardiovascular disease (CVD) is the leading global cause of death. Modifiable behavioral and psychosocial factors, particularly tobacco smoking and mental distress, significantly contribute to its pathogenesis and prognosis (van Trier et al. 2022).

Smoking is an established causal risk factor for CVD. Adjusted analyses from large cohorts show current smokers face markedly elevated risks of fatal cardiovascular events, with hazard ratios for cardiovascular mortality exceeding 2.5 in men and 3.8 in women compared to never‐smokers (Gallucci et al. 2020). Risks for acute myocardial infarction (AMI), cerebrovascular disease, and heart failure (HF) increase with smoking intensity and duration (Tasdighi et al. 2025). For instance, the Australian 45 and Up Study reported current smoking was associated with over twofold higher risks for these major CVD endpoints versus never‐smokers (Banks et al. 2019). A clear dose‐response relationship exists between pack‐years and CVD mortality. While cessation reduces risk, residual excess risk may persist long‐term.

Mental distress—encompassing symptoms of depression, anxiety, and stress—is also independently associated with adverse cardiovascular outcomes. Meta‐analyses indicate high distress levels confer about a 28% greater risk of CVD morbidity and mortality (Gaffey et al. 2022). This association persists after adjustment for traditional risk factors, including smoking. Proposed mechanisms involve both behavioral (e.g., increased smoking and physical inactivity) and physiological pathways (e.g., sympathetic activation, inflammation, and HPA‐axis dysregulation), which can accelerate atherosclerosis and thrombosis (A. Singh et al. 2025).

Randomized trial evidence on mental health interventions for hard CVD outcomes remains limited. However, meta‐analyses in cardiac patients suggest psychological therapies may reduce cardiac mortality by approximately 21%, indicating this risk domain may be modifiable (Nie et al. 2024). Although smoking and psychological distress are well‐established individual‐level risk factors for CVD, important gaps remain in understanding their population‐level relevance. Most prior studies have focused on individual behaviors and clinical cohorts, providing limited insight into how the prevalence and geographic clustering of these exposures shape regional and sociodemographic disparities in cardiovascular mortality. Moreover, smoking and mental distress frequently co‐occur and may jointly characterize adverse community risk environments, yet few studies have examined their combined distribution in relation to cause‐specific cardiovascular mortality. Evidence is particularly sparse regarding whether these associations differ across cardiovascular subtypes and demographic or urbanization strata at the population level. To address these gaps, we conducted a nationwide county‐level ecological analysis to evaluate the independent and joint population‐level associations of frequent mental distress and adult smoking prevalence with overall and cause‐specific cardiovascular mortality across the United States.

## 2 Materials and Methods

### 2.1 Study Design and Data Source

In this county‐level ecological study, we investigated the associations of mental distress and smoking with CVD mortality across the United States from 2016 to 2020. Demographic, socioeconomic, and comorbidity data were sourced from the County Health Rankings & Roadmaps program and the Centers for Disease Control and Prevention (CDC) Wide‐Ranging Online Data for Epidemiological Research (WONDER) platform (Siddiqi et al. 2022). As all analyzed variables were publicly available, institutional review board approval was not necessary.

### 2.2 Data Extraction and Mortality Outcome

Age‐adjusted mortality rates (AAMRs) (per 100,000 adults) for CVD–related deaths and specific CVD subcategories were calculated with 95% confidence intervals (CIs), based on the International Classification of Diseases, Tenth Revision (ICD‐10). CVD subtypes examined included ischemic heart disease (IHD) (I20–I25), AMI (I21–I22), HF (I50), and cerebrovascular disease (I60–I69) (Manemann et al. 2021). Data on death counts, population denominators, and demographic characteristics were obtained from national public health registries (CDC, n.d.). All analyses were stratified by age group (45–64 and ≥ 65 years), sex (male and female), race (White, American Indian or Alaska Native, and Black or African American), and degree of urbanization. Urban–rural classification followed the National Center for Health Statistics Urban–Rural Classification Scheme, which categorizes counties as large central metro, large fringe metro, medium metro, small metro, micropolitan, or noncore (Cross et al. 2020).

### 2.3 Frequent Mental Distress and Adult Smoking

Frequent mental distress was defined as the age‐adjusted proportion of adults reporting over 14 days of poor mental health in the past 30 days. County‐level estimates were obtained from the County Health Rankings & Roadmaps program, based on Behavioral Risk Factor Surveillance System (BRFSS) data (https://www.countyhealthrankings.org/health‐data/methodology‐and‐sources/data‐documentation/national‐data‐documentation‐2010‐2023). In accordance with the mortality outcome window, we used multi‐year averaged estimates corresponding to the 2016–2020 period, consistent with the methodology of the County Health Rankings national data documentation. To facilitate comparability across counties and to ensure consistent scaling between exposures, county‐level frequent mental distress and adult smoking prevalence were linearly rescaled to standardized indices ranging from 0 to 1. Specifically, for each variable, the standardized index was calculated by subtracting the minimum county‐level value observed nationally during the study period and dividing by the corresponding range (maximum − minimum). Under this transformation, a value of 0 represents the lowest observed county‐level prevalence, whereas a value of 1 represents the highest observed prevalence. Frequent mental distress was measured on a standardized scale ranging from 0 to 1, where higher values correspond to a greater burden of mental distress days. Counties were grouped into quartiles based on the national distribution of this index, from the lowest (0–0.25) to the highest (0.75–1.00).

Adult smoking prevalence was defined as the age‐adjusted percentage of adults reporting current cigarette smoking. Smoking data were likewise derived from County Health Rankings & Roadmaps and based on multi‐year BRFSS estimates aligned with the 2016–2020 study period (https://www.countyhealthrankings.org/health‐data/methodology‐and‐sources/data‐documentation/national‐data‐documentation‐2010‐2023). In this analysis, “tobacco” refers specifically to commercial tobacco products and does not include ceremonial or traditional uses. Smoking prevalence was also expressed as a standardized county‐level index ranging from 0 to 1, with higher scores indicating a greater proportion of adult smokers. Counties were then grouped into national quartiles based on this index, from the lowest (0–0.25) to the highest (0.75–1.00).

### 2.4 Covariates

To account for potential confounding, we adjusted for several established cardiovascular risk factors in both population stratification and multivariable models. Demographic covariates included the percentage of female residents, Hispanic residents, rural residents, and adults aged over 65 years. Data for other racial or ethnic groups were not available. Socioeconomic and healthcare‐related covariates consisted of the percentage of uninsured residents and household income inequality. We additionally adjusted for key lifestyle and metabolic risk factors, including obesity and diabetes prevalence.

### 2.5 Statistical Analysis

The analytic sample was restricted to counties with complete data for the three core variables: frequent mental distress, adult smoking prevalence, and CVD mortality. Counties missing any of these variables were excluded to strengthen the robustness and validity of the analyses. All covariates were fully observed with no missing data. A total of 3069 US counties—representing 97.6% of all counties nationwide—were retained for all subsequent analyses.

County‐level distributions of frequent mental distress, adult smoking prevalence, and CVD mortality were visualized using choropleth maps generated in R (ggplot2 package). CVD mortality was compared across quartiles of frequent mental distress and adult smoking using generalized linear models (GLMs). These comparisons were further stratified to estimate subgroup‐specific CVD mortality rates by demographic and urbanization categories.

Rate ratios (RRs) and 95% CIs were derived by comparing mortality rates between the highest and lowest quartiles of each exposure, for overall CVD and for the following subtypes: IHD, HF, AMI, and cerebrovascular disease. County‐level associations of frequent mental distress and adult smoking with CVD mortality were evaluated using GLMs with a quasi‐Poisson distribution. An offset term, the natural logarithm of the population size, was included to account for overdispersion and to appropriately model mortality rates. Models were fitted using the glm() function from the stats package in R (Kim et al. 2018).

Two sets of models were specified: Model 1 adjusted only for year and age group; Model 2 additionally adjusted for county‐level sociodemographic and health‐related covariates, including the proportions of female residents, Hispanic residents, rural population, and adults aged ≥ 65 years, percentage uninsured, household income inequality, obesity, and diabetes (Wang et al. 2025). We additionally assessed multicollinearity among covariates using variance inflation factor (VIF) diagnostics to evaluate potential violations of model assumptions (Table S1). Negative binomial regression models were fitted to assess the presence and extent of overdispersion in the count outcome and to evaluate whether allowing for an alternative variance structure materially altered the estimated associations (Tables S2 and S3). Furthermore, an additive model was developed to evaluate the presence of interaction. Additive interaction between adult smoking and frequent mental distress in relation to cardiovascular mortality was assessed by determining whether the combined effect of both exposures exceeded the sum of their individual effects. The additive interaction was quantified using the relative excess risk due to interaction (RERI), the attributable proportion due to interaction (AP), and the synergy index (S). The absence of additive interaction was indicated if the CI for RERI and AP included 0 and that for S included 1 (Table S4). Statistical significance was defined as 95% CIs excluding 1 or two‐sided *p*  < 0.05. All analyses were performed using R version 4.5.2.

## 3 Results

### 3.1 Frequent Mental Distress and CVD Mortality

**FIGURE 1:**
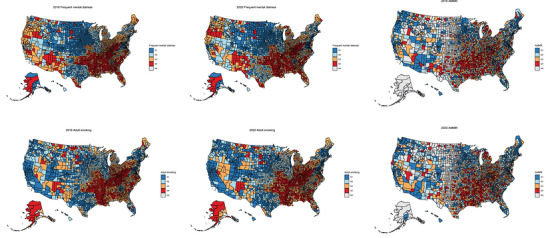
County‐level distribution of cardiovascular disease (CVD) mortality and related risk factors in the United States, 2016–2020. Choropleth maps show the geographic distribution of frequent mental distress (FMD), adult smoking prevalence, and age‐adjusted CVD mortality rates per 100,000 adults across US counties. Counties were categorized into quartiles (Q1–Q4) according to the distribution of each indicator, where Q denotes quartile. Darker colors represent higher values of the corresponding metric. Maps were generated using the ggplot2 package in R. This figure is descriptive in nature, and no formal statistical testing was performed.

In general, the southeastern and Appalachian regions of the United States exhibited the highest proportion of counties characterized by high frequency of mental distress, high prevalence of adult smoking, and increased AAMRs. In contrast, the western and northeastern regions showed lower levels of mental distress and smoking prevalence, corresponding to reduced AAMR across both 2016 and 2020 (Figure 1).

As shown in Table 1, the AAMR for total CVD increased progressively across quartiles of frequent mental distress, from 365.93 (95% CI 365.03–366.84) per 100,000 population in the first quartile to 509.24 (507.68–510.80) in the fourth quartile. A graded elevation in CVD mortality was consistently observed across all major sociodemographic strata, including age, sex, race, and urbanization level (all *p* < 0.001).

**TABLE 1:** Age‐adjusted mortality rates for CVD mortality stratified by frequent mental distress quartiles in US counties, 2016–2020.

Variables	Q1	Q2	Q3	Q4	Rate ratio (Q4 vs. Q1)	*p* value
All	365.93 (365.03, 366.84)	407.82 (406.99, 408.65)	448.76 (447.76, 449.77)	509.24 (507.68, 510.8)	1.61 (1.52, 1.7)	< 0.001
Age						
45–64	69.04 (68.54, 69.53)	92.22 (91.71, 92.74)	117.13 (116.44, 117.83)	154.89 (153.65, 156.14)	2.49 (2.4, 2.57)	< 0.001
≥ 65	887.62 (885.29, 889.96)	962.37 (960.27, 964.47)	1031.49 (1029.01, 1033.97)	1131.88 (1128.18, 1135.59)	1.26 (1.21, 1.32)	< 0.001
Sex						
Female	293.8 (292.71, 294.9)	324.85 (323.85, 325.85)	358.36 (357.13, 359.59)	397.79 (395.84, 399.75)	1.53 (1.42, 1.63)	< 0.001
Male	454.29 (452.68, 455.9)	510.28 (508.82, 511.75)	559.06 (557.28, 560.83)	636.77 (633.9, 639.65)	1.68 (1.59, 1.78)	< 0.001
Race						
Black	446.53 (442.42, 450.65)	489.87 (486.47, 493.28)	507.15 (503.63, 510.68)	544.83 (540.28, 549.4)	1.30 (1.25, 1.36)	< 0.001
White	371.81 (370.81, 372.82)	406.42 (405.52, 407.32)	443.51 (442.43, 444.6)	501.27 (499.52, 503.02)	1.59 (1.5, 1.69)	< 0.001
American Indian or Alaska Native	20.5 (16.41, 25.03)	70.43 (64.91, 76.19)	61.37 (55.64, 67.37)	177.38 (168.25, 186.74)	17.99 (14.57, 22.2)	< 0.001
Asian or Pacific Islander	239.08 (236.48, 241.69)	272.24 (268.97, 275.53)	236.76 (230.88, 242.71)	145.64 (136.66, 154.91)	0.49 (0.43, 0.57)	< 0.001
Urbanization						
Large Central Metro	361.08 (359.35, 362.81)	405.34 (403.98, 406.7)	436.68 (434.64, 438.73)	487 (483.73, 490.28)	1.42 (1.01, 2.01)	0.045
Large Fringe Metro	362.28 (360.94, 363.62)	414.75 (413.07, 416.44)	447.68 (445.23, 450.13)	491.29 (484.13, 498.49)	1.61 (1.2, 2.16)	0.002
Medium Metro	360.97 (358.57, 363.37)	387.49 (385.7, 389.28)	434.83 (433.07, 436.6)	467.5 (464.53, 470.48)	1.38 (1.18, 1.6)	< 0.001
Small Metro	392.29 (388.22, 396.39)	403.36 (400.33, 406.41)	451.6 (448.72, 454.49)	504.66 (500.73, 508.61)	1.13 (0.99, 1.31)	0.077
Micropolitan (Nonmetro)	400.38 (395.74, 405.03)	455.83 (451.69, 460)	502.82 (499.12, 506.53)	551.2 (547.1, 555.33)	1.06 (0.97, 1.17)	0.202
NonCore (Nonmetro)	399.26 (393.49, 405.07)	451.01 (446.08, 455.96)	493.31 (488.34, 498.3)	592.84 (587.78, 597.92)	1.25 (1.16, 1.35)	< 0.001

*Note*: Age‐adjusted mortality rates are expressed per 100,000 population with 95% confidence intervals (CIs). Counties were categorized into quartiles (Q1–Q4) based on the prevalence of frequent mental distress (FMD), defined as the percentage of adults reporting ≥ 14 days of poor mental health during the past 30 days. Rate ratios (RRs) and corresponding 95% CIs compare the highest quartile (Q4) with the lowest quartile (Q1). Mortality rates and RRs were estimated using generalized linear models with a quasi‐Poisson distribution and log‐transformed population size as an offset. Analyses were stratified by age group, sex, race, and urbanization level. Urbanization categories were defined according to the National Center for Health Statistics (NCHS) urban–rural classification scheme. *p* values were derived from two‐sided Wald tests.

Abbreviation: CVD, cardiovascular disease.

Age‐specific analysis revealed a steeper relative gradient among individuals aged 45–64 years. In this group, CVD mortality increased from 69.04 (68.54–69.53) in the first quartile to 154.89 (153.65–156.14) in the fourth quartile, yielding a RR of 2.49 (95% CI 2.40–2.57). Among adults aged over 65 years, mortality rose from 887.62 (885.29–889.96) to 1131.88 (1128.18–1135.59) across quartiles (RR 1.26; 95% CI 1.21–1.32). Monotonic increases were evident for both sexes, with males exhibiting higher absolute mortality rates in every quartile (Table 1).

Race‐stratified analyses demonstrated rising CVD mortality with increasing distress among White and Black populations. The RR comparing the Q4/Q1 was 1.59 (95% CI 1.50–1.69) for White individuals and 1.30 (1.25–1.36) for Black individuals (both *p* < 0.001). Notably, the gradient was substantially stronger among American Indian or Alaska Native populations (RR 17.99; 95% CI 14.57–22.20). Conversely, Asian or Pacific Islander populations exhibited an inverse association, with lower CVD mortality in the highest distress quartile (RR 0.49; 95% CI 0.43–0.57).

A positive association was generally present across urbanization levels. Statistically significant gradients were observed in large central metro, large fringe metro, medium metro, and noncore counties, whereas the associations were attenuated and non‐significant in small metro and micropolitan counties (Table 1).

**FIGURE 2:**
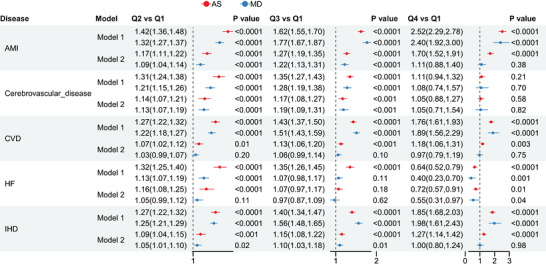
Associations of adult smoking (AS) and frequent mental distress (MD) with age‐adjusted mortality rates for cardiovascular disease (CVD) and its subtypes. Rate ratios (RRs) and 95% confidence intervals (CIs) were estimated using generalized linear models. Model 1 was adjusted for year and age group. Model 2 was further adjusted for county‐level sociodemographic and healthcare‐related characteristics, including the proportions of females, Hispanic residents, rural population, and adults aged ≥ 65 years; percentage of uninsured individuals; household income inequality; and the prevalences of obesity and diabetes. *p* values were derived from two‐sided Wald tests.

A graded dose‐response relationship was evident between county‐level frequent mental distress and mortality from major CVD subtypes (Table 2, Figure 2). The AAMR for IHD increased from 219.43 (95% CI: 218.72–220.14) to 317.98 (316.68–319.29) per 100,000 from the lowest to the highest distress quartile, corresponding to a relative risk of 1.68 (95% CI: 1.59–1.78). The steepest gradient was observed for AMI, with mortality rising from 58.11 (57.71–58.52) to 102.85 (101.97–103.72) (RR 1.87; 1.76–1.99). Cerebrovascular disease mortality also increased progressively, while the association with HF mortality was modest and non‐significant (RR 1.07; 0.98–1.17).

**TABLE 2:** Age‐adjusted mortality rates for CVD mortality and subtypes stratified by frequent mental distress quartiles in US counties, 2016–2020.

	Q1	Q2	Q3	Q4	Rate ratio (Q4 vs. Q1)	*p* value
CVD	365.93 (365.03, 366.84)	407.82 (406.99, 408.65)	448.76 (447.76, 449.77)	509.24 (507.68, 510.8)	1.61 (1.52, 1.7)	< 0.001
AMI	58.11 (57.71, 58.52)	66.52 (66.15, 66.9)	79.46 (78.97, 79.95)	102.85 (101.97, 103.72)	1.87 (1.76, 1.99)	< 0.001
Cerebrovascular disease	93.61 (93.12, 94.1)	101.88 (101.44, 102.33)	111.14 (110.59, 111.69)	119.04 (118.16, 119.93)	1.32 (1.22, 1.42)	< 0.001
HF	50.63 (50.26, 51)	55.23 (54.89, 55.57)	59.85 (59.43, 60.27)	65.65 (64.96, 66.35)	1.07 (0.98, 1.17)	0.158
IHD	219.43 (218.72, 220.14)	250.4 (249.74, 251.06)	276.95 (276.14, 277.76)	317.98 (316.68, 319.29)	1.68 (1.59, 1.78)	< 0.001

*Note*: Mortality rates are expressed per 100,000 population with 95% confidence intervals (CIs). Counties were classified into quartiles (Q1–Q4) according to the prevalence of frequent mental distress (FMD). Rate ratios (RRs) compare mortality rates between the highest and lowest quartiles (Q4 vs. Q1). Estimates were obtained using quasi‐Poisson generalized linear models with population size included as an offset. *p* values were calculated using two‐sided Wald tests.

Abbreviations: AMI, acute myocardial infarction; CVD, cardiovascular disease; HF, heart failure; IHD, ischemic heart disease.

Counties in higher quartiles of frequent mental distress exhibited progressively elevated cardiovascular mortality. Model 1 showed positive associations with cardiovascular mortality across several outcomes, particularly ischemic subtypes. Counties in higher distress quartiles experienced increased mortality for AMI, IHD, and overall CVD, with statistically significant associations observed across most exposure contrasts (all *p* < 0.001). In contrast, for cerebrovascular disease, the highest exposure contrast (Q4 vs. Q1) was not statistically significant (*p* = 0.70). In Model 2, the associations between mental distress and cardiovascular mortality were notably attenuated. For overall cardiovascular mortality, none of the exposure contrasts remained statistically significant after full adjustment (Q4 vs. Q1: *p* = 0.75). Statistically significant associations were observed only for AMI, cerebrovascular disease, and IHD at lower exposure levels (Q2 vs. Q1 and Q3 vs. Q1; all *p* < 0.05).

### 3.2 Adult Smoking and CVD Mortality

Counties with higher adult smoking prevalence generally demonstrated higher age‐adjusted CVD mortality. As presented in Table 3, the age‐adjusted total CVD mortality rate increased progressively across quartiles of adult smoking prevalence, from 376.45 (95% CI 375.78–377.12) per 100,000 population in the first quartile to 532.50 (530.75–534.25) in the fourth quartile. A monotonic dose–response relationship was observed across all sociodemographic subgroups (all *p* < 0.001).

**TABLE 3:** Age‐adjusted mortality rates for CVD mortality stratified by adult smoking prevalence quartiles in US counties, 2016–2020.

Variables	Q1	Q2	Q3	Q4	Rate ratio (Q4 vs. Q1)	*p* value
All	376.45 (375.78, 377.12)	424.12 (423.01, 425.22)	467.09 (465.9, 468.28)	532.5 (530.75, 534.25)	1.59 (1.51, 1.68)	< 0.001
Age						
45–64	77.43 (77.05, 77.82)	101 (100.27, 101.74)	126.12 (125.26, 126.98)	162.01 (160.63, 163.39)	2.27 (2.21, 2.34)	< 0.001
≥ 65	901.85 (900.14, 903.58)	991.88 (989.13, 994.64)	1066.23 (1063.32, 1069.14)	1183.5 (1179.34, 1187.67)	1.28 (1.23, 1.33)	< 0.001
Sex						
Female	303.41 (302.61, 304.22)	337.38 (336.02, 338.74)	372.04 (370.55, 373.52)	419.14 (416.92, 421.37)	1.61 (1.51, 1.72)	< 0.001
Male	468.32 (467.15, 469.49)	531.01 (529.05, 532.98)	588.89 (586.73, 591.05)	668.15 (664.87, 671.45)	1.62 (1.54, 1.71)	< 0.001
Race						
Black	472.86 (470.09, 475.64)	474.82 (470.36, 479.3)	518.23 (513.95, 522.53)	561.52 (556.51, 566.56)	1.3 (1.25, 1.34)	< 0.001
White	378.74 (378, 379.48)	421.32 (420.15, 422.5)	460.63 (459.34, 461.91)	524.24 (522.27, 526.21)	1.59 (1.51, 1.68)	< 0.001
American Indian or Alaska Native	71.98 (66.88, 77.26)	34.38 (29.61, 39.49)	56.44 (50.37, 62.86)	194.1 (183.59, 204.9)	4.04 (3.71, 4.41)	< 0.001
Asian or Pacific Islander	260.16 (258.09, 262.24)	210.46 (203.84, 217.19)	120.48 (113.32, 127.86)	84.13 (75.34, 93.4)	0.23 (0.18, 0.28)	< 0.001
Urbanization						
Large Central Metro	387.64 (386.54, 388.74)	423.24 (420.9, 425.59)	447.41 (444.54, 450.28)	505.11 (501.39, 508.83)	1.34 (0.98, 1.84)	0.067
Large Fringe Metro	373.46 (372.33, 374.6)	423.91 (421.42, 426.4)	457.26 (454.52, 460.01)	497.04 (491.16, 502.95)	1.42 (1.12, 1.79)	0.003
Medium Metro	361.38 (359.81, 362.96)	412.67 (410.65, 414.69)	454.42 (452.25, 456.59)	505.25 (501.34, 509.16)	1.39 (1.21, 1.6)	< 0.001
Small Metro	363.5 (360.41, 366.6)	422.12 (418.95, 425.3)	472.93 (469.74, 476.13)	523.34 (518.95, 527.75)	1.23 (1.09, 1.39)	0.001
Micropolitan (Nonmetro)	385.34 (380.96, 389.74)	451.99 (447.76, 456.23)	510.88 (507.23, 514.55)	562.59 (558.3, 566.89)	1.19 (1.08, 1.3)	< 0.001
NonCore (Nonmetro)	390.54 (384.94, 396.19)	453.57 (448.82, 458.34)	494.1 (489.2, 499.04)	603.93 (598.72, 609.16)	1.3 (1.2, 1.4)	< 0.001

*Note*: Age‐adjusted mortality rates are presented per 100,000 population with 95% confidence intervals (CIs). Counties were categorized into quartiles (Q1–Q4) according to adult smoking prevalence. Rate ratios (RRs) compare mortality rates in the highest quartile (Q4) with those in the lowest quartile (Q1). Estimates were derived from generalized linear models with a quasi‐Poisson distribution and log‐transformed population size as an offset. Stratified analyses were conducted by age group, sex, race, and urbanization level. Urbanization categories were defined based on the National Center for Health Statistics (NCHS) urban–rural classification scheme. *p* values were obtained using two‐sided Wald tests.

Abbreviation: CVD, cardiovascular disease.

Age‐stratified analyses indicated a stronger relative association among adults aged 45–64 years (RR 2.27, 95% CI 2.21–2.34) compared to those aged over 65 years (RR 1.28, 95% CI 1.23–1.33). Similar graded increases were observed in both sexes, with males showing consistently higher absolute mortality rates (Table 3).

Race‐stratified results showed increasing CVD mortality with higher smoking prevalence among White (RR 1.59, 95% CI 1.51–1.68) and Black (RR 1.30, 95% CI 1.25–1.34) populations. A pronounced gradient was observed among American Indian or Alaska Native populations (RR 4.04, 95% CI 3.71–4.41). In contrast, Asian or Pacific Islander populations exhibited an inverse association (RR 0.23, 95% CI 0.18–0.28).

Across urbanization strata, CVD mortality generally rose with increasing smoking prevalence. Statistically significant gradients were found in large fringe metro, medium metro, small metro, micropolitan, and noncore counties, whereas the association was not significant in large central metro counties (Table 3).

Table 4 and Figure 2 illustrate clear monotonic relationships between adult smoking prevalence and mortality from major CVD subtypes. The steepest gradient was observed for AMI (RR 1.97, 95% CI 1.86–2.07), followed by IHD (RR 1.60, 95% CI 1.52–1.69). Cerebrovascular disease mortality also rose progressively, whereas the association with HF was notably weaker (RR 1.11, 95% CI 1.02–1.21).

**TABLE 4:** Age‐adjusted mortality rates for CVD mortality and subtypes stratified by adult smoking prevalence quartiles in US counties, 2016–2020.

	Q1	Q2	Q3	Q4	Rate ratio (Q4 vs. Q1)	*p* value
CVD	376.45 (375.78, 377.12)	424.12 (423.01, 425.22)	467.09 (465.9, 468.28)	532.5 (530.75, 534.25)	1.59 (1.51, 1.68)	< 0.001
AMI	58.85 (58.56, 59.13)	74.63 (74.09, 75.18)	86 (85.36, 86.63)	118.85 (117.78, 119.92)	1.97 (1.86, 2.07)	< 0.001
Cerebrovascular disease	97.66 (97.3, 98.01)	103.41 (102.8, 104.01)	115.54 (114.86, 116.22)	120.72 (119.7, 121.74)	1.31 (1.21, 1.41)	< 0.001
HF	48.81 (48.55, 49.07)	57.26 (56.8, 57.73)	70.81 (70.26, 71.36)	74.13 (73.27, 74.99)	1.11 (1.02, 1.21)	0.013
IHD	231.03 (230.5, 231.56)	262.27 (261.37, 263.16)	282.37 (281.41, 283.34)	333.98 (332.5, 335.45)	1.60 (1.52, 1.69)	< 0.001

*Note*: Mortality rates are reported per 100,000 population with 95% confidence intervals (CIs). Counties were classified into quartiles (Q1–Q4) according to adult smoking prevalence. Rate ratios (RRs) compare mortality rates between the highest and lowest quartiles (Q4 vs. Q1). Estimates were generated using quasi‐Poisson generalized linear models with population size included as an offset. *p* values were calculated using two‐sided Wald tests.

Abbreviations: AMI, acute myocardial infarction; CVD, cardiovascular disease; HF, heart failure; and IHD, ischemic heart disease.

County‐level analysis revealed a consistent dose‐response pattern: higher smoking prevalence was associated with progressively increased mortality for all CVD outcomes. In Model 1, higher quartiles of adult smoking were consistently associated with increased mortality for AMI, IHD, HF, and overall CVD, with RRs increasing progressively from Q2 to Q4 compared with Q1 (all *p* < 0.0001). For cerebrovascular disease, modest associations were observed at lower exposure contrasts; however, the highest quartile comparison (Q4 vs. Q1) did not reach statistical significance (*p* = 0.21). After further adjustment for county‐level sociodemographic and health‐related factors in Model 2, associations for adult smoking were attenuated but largely remained robust. Elevated mortality risks persisted for AMI, IHD, and overall CVD, with Q4 vs. Q1 comparisons remaining statistically significant (all *p* < 0.0001). For cerebrovascular disease, associations were further attenuated and were no longer statistically significant across higher exposure levels (Q4 vs. Q1, *p* = 0.58). For HF, inverse associations emerged after full adjustment, with the highest smoking quartile remaining significantly associated with lower mortality (Q4 vs. Q1: *p* = 0.01). Overall, adult smoking prevalence demonstrated stable and consistent associations with cardiovascular mortality across models, despite multivariable adjustment.

In Table S1, we made VIF test to evaluate the adjusted factors in Model 2, and all confounding factors showed no multicollinearity. In Table S2, the negative binomial model yielded similar magnitude and direction of associations, indicating that overdispersion did not materially affect the estimated relationships.

Dispersion diagnostics indicated that the Poisson model was well specified, with a dispersion ratio close to 1 (0.9997, *p* = 0.912), suggesting no evidence of overdispersion. The negative binomial model showed significant underdispersion (dispersion ratio = 0.2198, *p* < 0.001). The full results of the dispersion tests are provided in Table S3.

### 3.3 Interactions Between Adults Smoking and Mental Distress on CVD Mortality

Results in Table S4 indicated a significant interaction between adult smoking prevalence and mental distress prevalence on CVD mortality. On the multiplicative scale, the interaction term was 0.97 (95% CI: 0.97–0.98). On the additive scale, the RERI was 0.24 (95% CI: 0.21–0.27), the AP was 0.09 (95% CI: 0.09–0.10), and the SI was 1.18 (95% CI: 1.17–1.19), indicating a positive additive interaction. In particular, the AP value of 0.09 suggests that approximately 9% of CVD mortality could be attributed to the interaction between adult smoking prevalence and mental distress prevalence. These findings suggest that counties with concurrently high smoking prevalence and high mental distress experience cardiovascular mortality rates that exceed expectations based on the sum of their individual associations.

## 4 Discussion

In this nationwide ecological analysis, counties with higher prevalence of frequent mental distress exhibited higher cardiovascular mortality rates, with the strongest correlations observed for overall CVD and ischemic subtypes. The persistence of significant associations for AMI and IHD after multivariable adjustment suggests that the population burden of mental distress is aligned with cardiovascular mortality patterns in the United States. The attenuation of estimates after adjustment—particularly for HF—likely reflects shared community‐level determinants between mental distress and other community‐level risk factors, such as socioeconomic disadvantage, behavioral risks, and comorbidity prevalence (Bober et al. 2025). Geographic clustering was notable, with high distress and high CVD mortality concentrated in the southeastern and Appalachian regions. These areas historically exhibit higher poverty rates, limited mental health care access, and lower insurance coverage (Wadhera and Joynt Maddox 2024). These structural disadvantages may jointly underlie elevated mental distress and cardiovascular mortality, resulting in spatial correlations that remain after statistical adjustment. Counties in western and northeastern states generally showed lower distress and CVD mortality, consistent with stronger public health infrastructure and broader service access (Cotton et al. 2025). Associations also varied across racial and ethnic groups. Markedly stronger associations among American Indian or Alaska Native populations may reflect cumulative impacts of historical marginalization, chronic stressors, and constrained healthcare access. Conversely, inverse associations observed among Asian or Pacific Islander populations may reflect differing sociocultural contexts, which may influence distress reporting, social cohesion, or underlying cardiovascular risk profiles (G. K. Singh et al. 2024). The particularly large RRs observed in counties with higher proportions of American Indian or Alaska Native residents warrant careful interpretation. At the ecological level, these estimates likely reflect the concentration of multiple structural and health disadvantages within the same geographic areas, including higher prevalence of smoking, greater psychosocial stress, limited access to healthcare services, and a higher burden of cardiometabolic comorbidities. In addition, the relatively small population size of American Indian or Alaska Native communities in many counties may contribute to greater variability in mortality rates, potentially amplifying relative estimates in ecological analyses. Importantly, these findings should not be interpreted as indicating elevated individual‐level biological risk, but rather as highlighting structural and contextual vulnerabilities that disproportionately affect these communities.

Adult smoking prevalence showed a stronger and more consistent graded association with cardiovascular mortality across all major subtypes, particularly for ischemic outcomes. Although adjustment attenuated relative risks, excess mortality remained significant, underscoring smoking prevalence as a key indicator of population‐level cardiovascular risk. The spatial distribution of smoking‐related CVD mortality overlapped with that of mental distress, with both concentrated in southeastern and Appalachian regions. These areas have historically experienced weaker tobacco control policies and limited cessation resources (Horn et al. 2022). Smoking prevalence at the county level likely reflects aggregated behavioral patterns as well as policy environments and cultural norms surrounding tobacco use. Urban–rural differences were also evident, with weaker gradients in large metropolitan counties, which may reflect greater behavioral heterogeneity and improved access to healthcare services (Calatayud and Moss 2024). Racial and ethnic disparities in smoking‐related mortality mirrored those for mental distress, with especially pronounced associations among American Indian or Alaska Native populations. These patterns align with higher smoking prevalence and structural barriers to preventive care in these communities (Patten et al. 2022).

In the broader context of nationwide studies investigating trends in mental health and health‐related behaviors, recent work by Balsara and colleagues analyzed BRFSS data from 2016 to 2022 and reported notable pandemic‐related disruptions in physical and mental health metrics as well as smoking prevalence across the United States. Their findings indicate persistent worsening of mental health indicators during the COVID‐19 period alongside reductions in smoking prevalence, highlighting evolving behavioral and psychosocial trends at the population level (Balsara et al. 2025). Our study, which focuses on county‐level frequent mental distress and adult smoking prevalence in relation to cardiovascular mortality prior to the pandemic period, complements these observations by providing evidence on how the spatial distribution of these risk factors across US counties is associated with mortality patterns. While the BRFSS trend analysis emphasizes temporal changes in mental health and smoking behaviors, our ecological analysis extends this line of inquiry by demonstrating spatial disparities and community‐level associations with cardiovascular outcomes, underscoring the importance of considering both temporal trends and geographic risk environments in public health planning and intervention strategies.

From a public health perspective, these findings highlight the importance of place‐based, population‐level interventions that jointly address tobacco use and mental distress within broader socioeconomic contexts. Given the pronounced clustering of high burden in specific regions, county‐ and state‐level health agencies could prioritize integrated tobacco control and mental health initiatives in high‐risk areas, particularly in rural and socioeconomically disadvantaged counties. Strengthening tobacco control remains a key actionable strategy and may include expanding comprehensive smoke‐free ordinances, increasing tobacco taxation, and investing in locally accessible cessation programs tailored to high‐prevalence communities. In parallel, improving the availability and affordability of mental health services—such as integrating mental health screening and referral into primary care and community health centers—may help address population‐level distress in underserved areas (Heetderks‐Fong and Bobb 2024; Bentley et al. 2025). Beyond the health sector, coordinated policies targeting upstream determinants, including housing instability, employment insecurity, and access to insurance coverage, may yield broader cardiovascular benefits. County‐level surveillance data, such as those used in this study, could be leveraged to identify priority regions, monitor progress over time, and guide resource allocation. Collectively, these targeted, data‐informed strategies underscore how public health, healthcare systems, and social policy can be aligned to reduce geographic disparities in cardiovascular mortality.

Several limitations should be considered when interpreting these findings. First, by design, this is an ecological analysis conducted at the county level. As such, the observed associations between frequent mental distress, adult smoking prevalence, and cardiovascular mortality reflect population‐level correlations and should not be interpreted as evidence of individual‐level risk. The potential for ecological fallacy cannot be excluded, as individuals experiencing mental distress or smoking within a given county are not necessarily the same individuals contributing to cardiovascular mortality outcomes. Second, residual confounding is likely, as county‐level measures cannot fully capture individual behaviors or clinical factors. Nevertheless, persistent associations after extensive adjustment suggest that distress and smoking prevalence reflect meaningful community‐level risk environments. In addition, formal sensitivity analyses were not feasible because the exposure variables were obtained as fixed, pre‐aggregated county‐level estimates from publicly available data sources. This data structure limited the ability to apply alternative exposure definitions, categorizations, or temporal specifications. However, detailed documentation of data sources, variable construction, and model specifications is provided to support transparency and reproducibility. In addition, our study period included data from 2020, during which the COVID‐19 pandemic may have influenced mental distress, smoking behaviors, healthcare utilization, and cardiovascular mortality. Although the exposure variables were based on multi‐year averaged county‐level estimates that may reduce short‐term fluctuations, potential pandemic‐related effects cannot be completely excluded. Therefore, the findings should be interpreted with appropriate caution. Finally, the cross‐sectional design limits causal interpretation, underscoring the need for longitudinal and quasi‐experimental studies to assess how changes in policies and social conditions affect cardiovascular mortality over time.

## 5 Conclusion

This nationwide ecological study found that higher county‐level prevalence of frequent mental distress and adult smoking was associated with increased cardiovascular mortality, particularly for IHD and AMI. These associations vary by region and demographic group, with the highest burdens concentrated in socioeconomically disadvantaged areas. The results suggest that mental distress and smoking prevalence are salient indicators of community‐level cardiovascular risk. Integrated public health strategies that simultaneously address tobacco control, mental health, and underlying social determinants are warranted to reduce geographic and socioeconomic disparities in cardiovascular mortality.

## Author Contributions

**Yu‐Jun Xiong**: methodology, validation, software. **Shengpang Wang**: conceptualization, investigation, writing – original draft. **Tian Lv**: writing – review and editing, investigation, project administration. **Xiang‐Da Meng**: formal analysis, project administration, data curation. **Jingjing Lou**: writing – review and editing, formal analysis, supervision, resources.

## Funding

The authors have nothing to report.

## Ethics Statement

The analysis was based on de‐identified, publicly accessible data from CDC WONDER, and thus ethical approval was not required.

## Consent

The authors have nothing to report.

## Conflicts of Interest

The authors declared no conflicts of interest.

## Supporting information




**Supplementary Tables**: brb371590‐sup‐0001‐TableS1‐S4.docx

## Data Availability

The raw data supporting the conclusions of this article can be found here: https://wonder.cdc.gov/ and https://www.countyhealthrankings.org/explore‐health‐rankings/rankings‐data‐documentation/national‐data‐documentation‐2010‐2019. The R scripts used for statistical analyses are available from the corresponding author upon reasonable request.

## References

[brb371590-bib-0001] Balsara, K. , V. Kotharkar , P. Galiatsatos , and N. Kanarek . 2025. “Disruption and Recovery in Physical and Mental Health, Body Mass Index and Smoking During the COVID‐19 Pandemic: A Trend Analysis of US BRFSS Data From 2016 to 2022.” BMJ Public Health 3, no. 2: e002765. 10.1136/bmjph-2025-002765. 41040506 PMC12487425

[brb371590-bib-0002] Banks, E. , G. Joshy , R. J. Korda , et al. 2019. “Tobacco Smoking and Risk of 36 Cardiovascular Disease Subtypes: Fatal and Non‐Fatal Outcomes in a Large Prospective Australian Study.” BMC Medicine 17, no. 1: 128. 10.1186/s12916-019-1351-4. 31266500 PMC6607519

[brb371590-bib-0003] Bentley, R. , K. Mason , D. Jacobs , et al. 2025. “Housing as a Social Determinant of Health: A Contemporary Framework.” The Lancet Public Health 10, no. 10: e855–e864. 10.1016/S2468-2667(25)00142-2. 40953578

[brb371590-bib-0004] Bober, T. , E. N. Guhl , S. Rothenberger , et al. 2025. “Impact of Neighborhood Factors on Heart Failure Outcomes.” JACC: Advances 4, no. 10 pt. 2: 102146. 10.1016/j.jacadv.2025.102146. 40953541 PMC12464708

[brb371590-bib-0005] Calatayud, B. M. , and J. L. Moss . 2024. “Rural/Urban Differences in Uptake of Preventive Healthcare Services: Variability in Observed Relationships Across Measures of Rurality.” Journal of Public Health Research 13, no. 1: 22799036241238670. 10.1177/22799036241238670. 38505764 PMC10949549

[brb371590-bib-0006] CDC . n.d. “Underlying Cause of Death 1999–2020.” https://wonder.cdc.gov/wonder/help/ucd.html.

[brb371590-bib-0007] Cotton, A. , P. R. Salerno , S. V. Deo , et al. 2025. “The Association Between County‐Level Social Determinants of Health and Cardio‐Kidney‐Metabolic Disease Attributed All‐Cause Mortality in the US: A Cross Sectional Analysis.” The American Journal of the Medical Sciences 369, no. 4: 491–497. 10.1016/j.amjms.2025.01.007. 39848403 PMC12046418

[brb371590-bib-0008] Cross, S. H. , M. R. Mehra , D. L. Bhatt , et al. 2020. “Rural‐Urban Differences in Cardiovascular Mortality in the US, 1999‐2017.” JAMA 323, no. 18: 1852. 10.1001/jama.2020.2047. 32396176 PMC7218488

[brb371590-bib-0009] Gaffey, A. E. , E. C. Gathright , L. M. Fletcher , and C. M. Goldstein . 2022. “Screening for Psychological Distress and Risk of Cardiovascular Disease and Related Mortality.” Journal of Cardiopulmonary Rehabilitation and Prevention 42, no. 6: 404–415. 10.1097/HCR.0000000000000751. 36342683 PMC9646240

[brb371590-bib-0010] Gallucci, G. , A. Tartarone , R. Lerose , A. V. Lalinga , and A. M. Capobianco . 2020. “Cardiovascular Risk of Smoking and Benefits of Smoking Cessation.” Journal of Thoracic Disease 12, no. 7: 3866–3876. 10.21037/jtd.2020.02.47. 32802468 PMC7399440

[brb371590-bib-0011] Heetderks‐Fong, E. , and A. Bobb . 2024. “Community Mental Health Workers: Their Workplaces, Roles, and Impact.” Community Mental Health Journal 60, no. 8: 1547–1556. 10.1007/s10597-024-01306-2. 38896213

[brb371590-bib-0012] Horn, K. , N. Schoenberg , S. Rose , K. Romm , and C. J. Berg . 2022. “Tobacco Use Among Appalachian Adolescents: An Urgent Need for Virtual Scale out of Effective Interventions.” Tobacco Prevention & Cessation 8: 1–10. 10.18332/tpc/155331. PMC9635399 36404952

[brb371590-bib-0013] Kim, J. , Y. Zhang , J. Day , and H. Zhou . 2018. “MGLM: An R Package for Multivariate Categorical Data Analysis.” The R Journal 10, no. 1: 73. 10.32614/RJ-2018-015. 32523781 PMC7286576

[brb371590-bib-0014] Manemann, S. M. , Y. Gerber , S. J. Bielinski , et al. 2021. “Recent Trends in Cardiovascular Disease Deaths: A State Specific Perspective.” BMC Public Health 21, no. 1: 1031. 10.1186/s12889-021-11072-5. 34074276 PMC8169395

[brb371590-bib-0015] Nie, Y. , N. Wang , M. Chi , et al. 2024. “Effects of Psychological Interventions on Clinical Outcomes in Patients With Cardiovascular Diseases: A Systematic Review and Meta‐Analysis.” Journal of Psychosomatic Research 187: 111938. 10.1016/j.jpsychores.2024.111938. 39321711

[brb371590-bib-0016] Patten, C. A. , V. Y. Hiratsuka , S. H. Nash , et al. 2022. “Smoking Patterns among Urban Alaska Native and American Indian Adults: The Alaska EARTH 10‐Year Follow‐Up Study.” Nicotine & Tobacco Research 24, no. 6: 840–846. 10.1093/ntr/ntab245. 34850172 PMC9048910

[brb371590-bib-0017] Siddiqi, T. J. , A. M. Khan Minhas , S. J. Greene , et al. 2022. “Trends in Heart Failure‐Related Mortality Among Older Adults in the United States From 1999‐2019.” JACC: Heart Failure 10, no. 11: 851–859. 10.1016/j.jchf.2022.06.012. 36328654

[brb371590-bib-0018] Singh, A. , R. Riaz , A. Verma , et al. 2025. “Integrating Mental Health and Cardiovascular Wellness: Synergistic Impacts and the Promise of Comprehensive Care Models.” Annals of Medicine & Surgery 87, no. 8: 4963–4974. 10.1097/MS9.0000000000003391. 40787495 PMC12333827

[brb371590-bib-0019] Singh, G. K. , H. Lee , L. H. Kim , and S. D. Williams . 2024. “Social Determinants of Health Among American Indians and Alaska Natives and Tribal Communities: Comparison With Other Major Racial and Ethnic Groups in the United States, 1990–2022.” International Journal of Maternal and Child Health and AIDS 13: e010. 10.25259/IJMA_10_2024. 38840933 PMC11152578

[brb371590-bib-0020] Tasdighi, E. , Z. Yao , Z. A. Dardari , et al. 2025. “Association Between Cigarette Smoking Status, Intensity, and Cessation Duration With Long‐Term Incidence of Nine Cardiovascular and Mortality Outcomes: The Cross‐Cohort Collaboration (CCC).” PLOS Medicine 22, no. 11: e1004561. 10.1371/journal.pmed.1004561. 41252354 PMC12626310

[brb371590-bib-0021] van Trier, T. J. , N. Mohammadnia , M. Snaterse , R. J. G. Peters , H. T. Jørstad , and W. A. Bax . 2022. “Lifestyle Management to Prevent Atherosclerotic Cardiovascular Disease: Evidence and Challenges.” Netherlands Heart Journal 30, no. 1: 3–14. 34762283 10.1007/s12471-021-01642-y PMC8724344

[brb371590-bib-0022] Wadhera, R. K. , and K. E. Joynt Maddox . 2024. “Policy Strategies to Advance Cardiovascular Health in the United States—Building on a Century of Progress.” Circulation: Cardiovascular Quality and Outcomes 17, no. 4:e010149. 10.1161/CIRCOUTCOMES.123.010149. 38626057

[brb371590-bib-0023] Wang, X. , Y. Gu , Y. Wang , et al. 2025. “The Role of Environmental Access to Exercise Opportunities in Cardiovascular Mortality: Evidence From a Nationwide Study.” BMC Medicine 23, no. 1: 228. 10.1186/s12916-025-04060-8. 40251637 PMC12008913

