# Supplementary material for: Mental Distress and Smoking in Relation to Cardiovascular Mortality in the United States Population
Source: Brain Behav. 2026 Jul 9;16(7):e71590. doi: 10.1002/brb3.71590 (PMC13347143; doi:10.1002/brb3.71590)
Supplement: Supplementary file 1 — Supplementary Tables: brb371590‐sup‐0001‐TableS1‐S4.docx [file BRB3-16-e71590-s001.docx]

Supplementary Table 1 Variance Inflation Factor test of model 2 in multivariable-adjusted logistic regression analysis.

|  | GVIF |
| --- | --- |
| Adult smoking | 3.484714551 |
| Mental distress | 3.042445636 |
| The proportions of female residents | 1.263448948 |
| The proportions of Hispanic residents | 1.855544215 |
| The proportions of rural residents | 1.921261497 |
| The proportions of residents aged over 65 | 1.546111304 |
| The proportions of uninsured residents | 1.361162867 |
| household income inequality | 1.811709181 |
| Obesity | 2.018472351 |
| Diabetes Mellitus | 2.347968407 |

Supplementary Table 2 Negative binomial models of adult smoking and mental distress on CVD mortality.

| Parameter | IRR | 2.5% | 97.5% |
| --- | --- | --- | --- |
| (Intercept) | 0.0000293358 | 0.0000231737 | 0.00003717219 |
| Adult smoking | 1.0226570429 | 1.0183750873 | 1.02695671863 |
| Mental distress | 1.0627681112 | 1.0544215703 | 1.07118441370 |

Supplementary Table 3 Dispersion ratio of Poisson and negative binomial models.

| Model | Dispersion Ratio | *P* value |
| --- | --- | --- |
| Poisson model | 0.9997 | 0.912 |
| Negative binomial model | 0.2198 | <0.001 |

Supplementary Table 4 Interaction analysis of adult smoking prevalence and mental distress prevalence on CVD mortality.

| Variables | HR [95% CI] |
| --- | --- |
| Multiplicative scale | 0.97 [0.97, 0.98] |
| RERI | 0.24 [0.21, 0.27] |
| AP | 0.09 [0.09, 0.1] |
| SI | 1.18 [1.17, 1.19] |
